# EZH2 inhibitors promote β-like cell regeneration in young and adult type 1 diabetes donors

**DOI:** 10.1038/s41392-023-01707-x

**Published:** 2024-01-01

**Authors:** Keith Al-Hasani, Safiya Naina Marikar, Harikrishnan Kaipananickal, Scott Maxwell, Jun Okabe, Ishant Khurana, Thomas Karagiannis, Julia J. Liang, Lina Mariana, Thomas Loudovaris, Thomas Kay, Assam El-Osta

**Affiliations:** 1 Epigenetics in Human Health and Disease Program, Baker Heart and Diabetes Institute, 75 Commercial Road, Melbourne, 3004 VIC Australia; 2 Department of Diabetes, Central Clinical School, Monash University, Melbourne, 3004 VIC Australia; 3 Epigenetics in Human Health and Disease Laboratory, Central Clinical School, Monash University, Melbourne, 3004 VIC Australia; 4 School of Science, STEM College, RMIT University, Melbourne, 3001 VIC Australia; 5 Immunology and Diabetes Unit, St Vincent’s Institute of Medical Research, Fitzroy, 3065 VIC Australia; 6 Department of Medicine and Therapeutics, The Chinese University of Hong Kong, Sha Tin, Hong Kong SAR; 7 Hong Kong Institute of Diabetes and Obesity, Prince of Wales Hospital, The Chinese University of Hong Kong, 3/F Lui Che Woo Clinical Sciences Building, 30-32- Ngan Shing Street, Sha Tin, Hong Kong SAR; 8 Li Ka Shing Institute of Health Sciences, The Chinese University of Hong Kong, Sha Tin, Hong Kong SAR; 9 Biomedical Laboratory Science, Department of Technology, Faculty of Health, University College Copenhagen, Copenhagen, Denmark

**Keywords:** Endocrine system and metabolic diseases, Preclinical research

## Abstract

β-cells are a type of endocrine cell found in pancreatic islets that synthesize, store and release insulin. In type 1 diabetes (T1D), T-cells of the immune system selectively destroy the insulin-producing β-cells. Destruction of these cells leads to a lifelong dependence on exogenous insulin administration for survival. Consequently, there is an urgent need to identify novel therapies that stimulate β-cell growth and induce β-cell function. We and others have shown that pancreatic ductal progenitor cells are a promising source for regenerating β-cells for T1D owing to their inherent differentiation capacity. Default transcriptional suppression is refractory to exocrine reaction and tightly controls the regenerative potential by the EZH2 methyltransferase. In the present study, we show that transient stimulation of exocrine cells, derived from juvenile and adult T1D donors to the FDA-approved EZH2 inhibitors GSK126 and Tazemetostat (Taz) influence a phenotypic shift towards a β-like cell identity. The transition from repressed to permissive chromatin states are dependent on bivalent H3K27me3 and H3K4me3 chromatin modification. Targeting EZH2 is fundamental to β-cell regenerative potential. Reprogrammed pancreatic ductal cells exhibit insulin production and secretion in response to a physiological glucose challenge ex vivo. These pre-clinical studies underscore the potential of small molecule inhibitors as novel modulators of ductal progenitor differentiation and a promising new approach for the restoration of β-like cell function.

## Introduction

Diabetes is a global disease affecting approximately 400 million people worldwide and is responsible for 9.9% of all-cause mortality. The destruction of a functional insulin-producing β-cell mass in the islets of Langerhans of the pancreas leads to an inability to correctly regulate blood glucose levels and is associated with the development of insulin-dependent diabetes. While current pharmaceutical options for diabetes treatment help control blood glucose levels they do not prevent, retard or reverse the decline in insulin-secreting β-cells.^1,2^

Present treatments designed to address T1D centre around the restoration of impaired β-cell mass in diabetic individuals, utilising methods such as whole pancreas or islet transplantation.^1^ While these approaches have demonstrated clinical effectiveness, they encounter the significant challenge of a shortage of available donors, coupled with the potential adverse effects linked to immunosuppressive medications.^3^ Thus, there is an urgent need to identify novel therapies that stimulate growth and induce β-cell function. The existence of ductal progenitors present in a stem cell niche within the pancreas has widely been debated. Initial observational studies that showed the clustering of islets near the ductal epithelium led to the belief that endocrine cells were derived from these so-called progenitors within the pancreatic ducts.^4–7^ Pancreatic injury models, including ductal ligation and partial pancreatomies, supported the idea that these cells in the ductal niche can develop into islet endocrine cells upon NGN3 expression,^8^ mirroring embryonic development.^9^ However, key lineage tracing experiments show a lack of adult β-cells emerging from ductal cells,^10^ leading to the abandonment of the pancreatic ductal progenitor model, in favour of self-replicating β-cells maintaining the adult populations albeit at low levels.

Since then, numerous studies have shown conflicting results, both in favour,^11,12^ and against the existence of the pancreatic progenitors in adults using various injury models,^13–15^ transgenic models^16^ and pharmacological treatments to induce the differentiation of β-cells from progenitors.^17,18^ Recent studies have shown that ductal *NGN3*+ cells can differentiate into adult β-cells^19^ and is consistent with single-cell RNA sequencing studies of the ductal progenitor niche.^20^ These findings align with evidence that ductal progenitors can become α-cells and then evolve into β-cells through the overexpression of Pax4 or downregulation of Arx, within the context of α-cell trans-differentiation.^21,22^

A case report has recently shown that it is now possible to partly restore insulin gene expression from pancreatic ductal cells by converting the refractory nature of chromatin using GSK126, an FDA-approved EZH2 inhibitor.^23^ Despite showing the β-cell-like conversion of exocrine cells from a T1D donor with absolute β-cell destruction, residual doubts remained on the generalizability of the *n* = 1 finding. Moreover, questions persist on the significance of default suppression and whether reducing H3K27me3 to restore gene expression are sufficient to influence protein expression in situ. The evidence to date is based on the supposition that EZH2 inhibition would support functional insulin secretion that would reflect the regulatory events in the pancreas. The death of a juvenile with newly diagnosed T1D along with a long-term adult T1D and a healthy non-diabetic, prompted our examination of pancreatic ductal cells using GSK126 to identify metabolic β-like capacity. Furthermore, to characterise regenerative outcomes, we subsequently evaluated Tazemetostat, a selective-competitive inhibitor of EZH2. Approved by the FDA in Jan 2020 under the brand name Tazverik, this drug is used for the treatment of adults and adolescents with sarcoma. The aim of this study was to characterise the influence of these small molecule inhibitors on the regenerative capacity to better understand default transcriptional suppression by the histone methyltransferase in the diabetic pancreas. We show that 48 h of stimulation with EZH2 inhibitors is sufficient to restore key β-cell indices not just limited to transcriptional activation, but also expression and secretion of insulin from a primary pancreatic exocrine milieu.

## Results

To investigate the reactivation of pancreatic progenitor cells, we assessed the small molecule inhibitors GSK126 and Tazemetostat (Taz) for regenerative β-cell capacity following surgical resection of human pancreatic tissues from three donors.

### Molecular modelling of GSK126 and Tazemetostat bound to the EZH2 methyltransferase

**Fig. 1:**
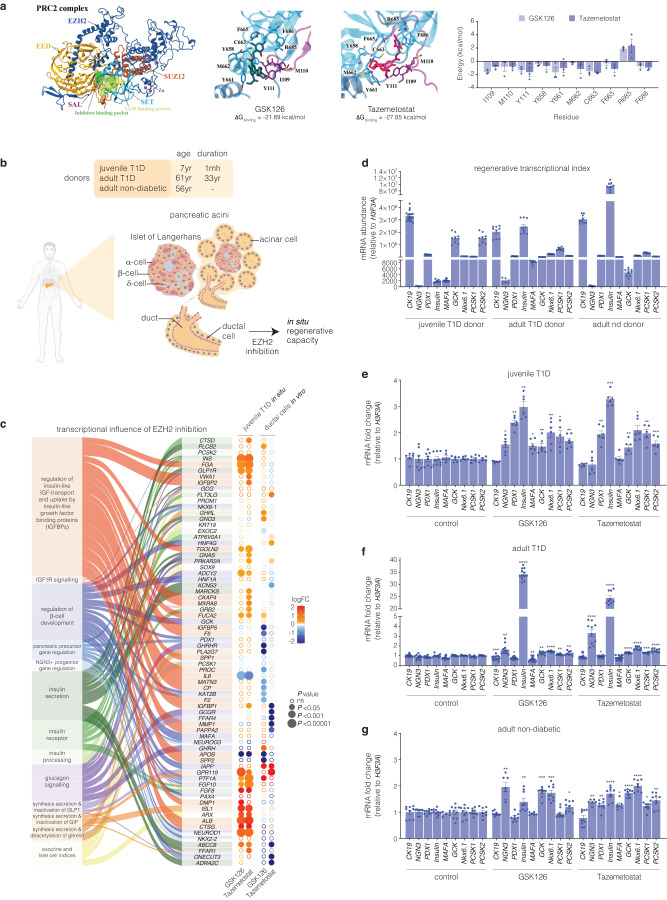
Inhibition of EZH2 by GSK126 and Tazemetostat reactivates the expression of endocrine markers in exocrine cells. **a** Structure of the human polycomb repressive 2 (PRC2) complex composed of EZH2 (dark blue), EED (light orange), and SUZ12 (dark orange) displayed in cartoon representation. The catalytic SET domain (cyan) and the SET activation loop (SAL, purple) of EZH2 are highlighted. The binding pocket for pyridine inhibitors partially overlaps with the SAM binding pocket, shown in surface representation. Binding free energies of GSK126 and Taz were calculated with MM-PBSA. Binding energy was decomposed on a per-residue basis, with energies for residues of the SET and SAL domains displayed as mean ± SEM. Structures of GSK126 (teal), Taz (magenta), and residues of the SET (cyan) and SAL (purple) domains are shown as sticks. **b** Schematic of human exocrine tissue isolation from Type 1 diabetic (T1D) and non-diabetic donors featuring the in vivo location of ductal cells stimulated by EZH2 inhibitors. **c** RNA-seq analysis showing differential expression of canonical β-cell and exocrine markers derived from Reactome database in T1D pancreatic tissue and human pancreatic ductal epithelial cells following EZH2 inhibition using GSK126 or Taz. The left panel illustrates the association of functional pathway descriptors with individual genes. The right panel displays differential gene expression by inhibitor group in circular format. Log2 fold change (logFC) is represented by a diverging red (increase) – blue (decrease) colour gradients. Expression significance (decreasing *P*-value) is illustrated by larger circular diameter. Hollow circles are non-significant change (ns = *P* > 0.05). **d** Comparison of mRNA expression levels of key regenerative genes that include *CK19, NGN3, PDX1*, *INS*, *MAFA, GCK, NKX6.1, PCSK1* and *PCSK2* relative to *H3F3A* in T1D and non-diabetic donors before EZH2 inhibitor stimulation. Insulin (*INS*) expression is barely detectable in juvenile T1D and significantly reduced in adult T1D donor when compared to the non-diabetic donor. Data are represented as mean of experiments performed using non-diabetic and T1D donors with 3 technical replicates, error bars are S.E.M. **e** Fold change in the transcriptional expression index of *CK19, NGN3, PDX1, INS, MAFA, GCK, NKX6.1, PCSK1*, and *PCSK2* relative to *H3F3A* mRNA in juvenile T1D donor. Data are represented as mean of experiments conducted in non-diabetic and T1D donors. EZH2 inhibition studies were repeated 3 times with technical replicates. Statistical significance was calculated by comparing control vs inhibitor values using Student *t*-test, *P < 0.05, **P < 0.01, ***P < 0.001, error bars are S.E.M. **f** Fold change in the transcriptional expression index of *CK19, NGN3, PDX1, INS, MAFA, GCK, NKX6.1, PCSK1* and *PCSK2* in adult T1D donor, displayed as fold change relative to *H3F3A* mRNA levels. Data are represented as mean of experiments conducted in non-diabetic and T1D donors. EZH2 inhibition studies were repeated repeated 3 times with technical replicates. Statistical significance was calculated by comparing control vs inhibitor values using Student *t*-test, *P < 0.05, **P < 0.01, ***P < 0.001, ****P < 0.0001, error bars are S.E.M. **g** Fold change in the transcriptional expression index of *CK19, NGN3, PDX1, INS, MAFA, GCK, NKX6.1, PCSK1* and *PCSK2* in adult non-diabetic donor, shown as fold change relative to *H3F3A* mRNA levels. Data are represented as mean of experiments conducted in non-diabetic and T1D donors. EZH2 inhibition studies were repeated repeated 3 times with technical replicates. Statistical significance was calculated by comparing control vs inhibitor values using Student *t*-test, *P < 0.05, **P < 0.01, ***P < 0.001, ****P < 0.0001, error bars are S.E.M

To investigate the structural influences on binding to the catalytic domains of the EZH2 protein, predictive molecular modelling studies were performed. We report the small molecule inhibitors bind EZH2 with Taz showing a higher binding affinity at the catalytic domain (Fig. 1a). Molecular dynamics simulations show strong energy contributions from Y661 and C663 in the catalytic SET domain and I109 and Y111 in the SAL region of EZH2. Residues that strongly contribute to SET domain binding that include C663, F665 and F686 are also involved in binding of the SAH/SAM cofactor^24^ indicative of competitive binding by GSK126 and Taz.

### Transcriptome analysis of human pancreatic ductal cells in response to EZH2 inhibition

Ex vivo exocrine tissue was obtained from 3 donors of varying age and diabetic status (Fig. 1b). To identify differentially expressed genes and pathways responsive to pharmacological inhibition of EZH2, we performed RNA sequencing to generate transcriptome profiles from exocrine cells derived from a juvenile Type 1 Diabetes (T1D) donor including pancreatic ductal epithelial cells following stimulation of GSK126 or Taz for 48 h. Figure 1c shows gene signatures across exocrine and β-cell pathways that converge on networks that are central to β-cell neogenesis. Analysis of juvenile T1D exocrine cells stimulated with EZH2 inhibitors show upregulation of genes such as *INS*, *NEUROD1*, *FGF10*, *PTF1A* and *IAPP*. We also observe trends in transcriptional expression indices (TEI) for pancreatic β-cell maturation that include *PAX4*, *NKX6-1*, *NKX2-2*, *KAT2B*, *ONECUT*, *HNF1A*, *GCK*, *MAFA* and *PDX1*.^25^ Furthermore, we observe strong correspondence with *NGN3*+ progenitor and pancreatic precursor pathways with transcriptional expression of *FGF10, PTF1A, NKX6-1, NKX2-2, KAT2B, PDX1, PAX4* and *ONECUT3* indicative of endocrine progenitor specification and β-cell development.^26^ Differential expression in the IGF pathway genes such as *FGA, VWA1, TGOLN2, ALB, CTSG, IL6, APOB*, and *FUCA2* indicate regulatory roles in IGF signalling essential for β-cell proliferation and survival. IGF1R genes are pivotal for β-cell development, driving both proliferation and anti-apoptotic effects.^27^ Furthermore, transcriptional changes are observed for key genes in the insulin/glucagon and incretin pathways (*GLP1R, INS, FGF10, FGF8, ABCC8, ISL1, ADCY2*). These studies suggest GSK126 and Taz influence the expression of genes involved in glucose metabolism and exocrine hormone regulation.

In addition to the transcriptome profiling studies, we also assessed key genes by qRT-PCR from pancreatic ductal cells derived from juvenile and adult T1D donors. The regenerative TEI were compared to pancreatic ductal cells derived from a non-diabetic adult (Fig. 1d). Subsequent evaluation of CK19+ve cells derived from juvenile T1D (Fig. 1e), adult T1D (Fig. 1f) and adult non-diabetic (Fig. 1g) donors show pharmacological EZH2 inhibition influences the transcriptional expression of endocrine markers. Moreover, stimulation of ductal cells from insulin-dependent T1D donors with GSK126 or Taz influenced insulin (*INS*) mRNA expression including the key gene, *PDX1*, crucial for preserving β-cell identity.

### Refractory H3K27me3 content of endocrine genes are reduced in exocrine tissue following EZH2 inhibition

**Fig. 2:**
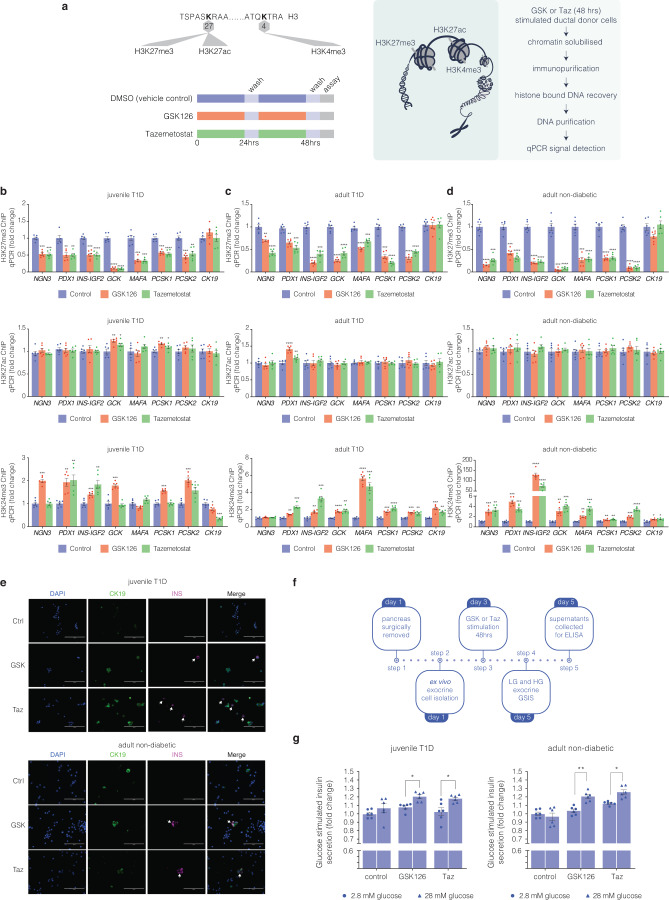
Bivalent chromatin protects regenerative exocrine capacity and insulin expression from default transcriptional suppression. **a** Schematic of histone tail modification for H3K27me3, H3K27ac and H3K4me3 content. Also shown is the protocol used to stimulate CK19+ve ductal cells derived from juvenile and adult T1D donors with EZH2 inhibitors for 48 h and assessed for chromatin content, immunofluorescence and GSIS assays. **b** GSK126 and Taz influences bivalent chromatin domains in human exocrine CK19+ve cells derived from juvenile T1D donor. Quantitative PCR analyses of DNA in ChIP using anti-H3K27me3, anti-H3K27ac and anti-H3K4me3 antibodies for *NGN3, PDX1, INS-IGF2, MAFA, GCK, PCSK1, PCSK2*, and *CK19* are displayed as fold change calculated and adjusted to control values. Data are represented as mean ± S.E.M. of percent input (EZH2 inhibition; *n* = 6). Vehicle control was DMSO. Statistical significance was calculated by comparing control vs GSK126 or Tazemetostat using Student *t*-test, *P < 0.05, **P < 0.01, ***P < 0.001, ****P < 0.0001. **c** GSK126 and Taz influences bivalent chromatin domains in human exocrine CK19+ve cells derived from adult T1D donor. Quantitative PCR analyses of DNA in ChIP using anti-H3K27me3, anti-H3K27ac and anti-H3K4me3 antibodies for *NGN3, PDX1, INS-IGF2, MAFA, GCK, PCSK1, PCSK2*, and *CK19* are displayed as fold change calculated and adjusted to control values. Data are represented as mean ± S.E.M. of percent input (EZH2 inhibition; *n* = 6). Vehicle control was DMSO. Statistical significance was calculated by comparing control vs GSK126 or Tazemetostat using Student *t*-test, **P < 0.01, ***P < 0.001, ****P < 0.0001. **d** GSK126 and Taz influences bivalent chromatin domains in human exocrine CK19+ve cells derived from adult non-diabetic donor. Quantitative PCR analyses of DNA in ChIP using anti-H3K27me3, anti-H3K27ac and anti-H3K4me3 antibodies for *NGN3, PDX1, INS-IGF2, MAFA, GCK, PCSK1, PCSK2*, and *CK19* are displayed as fold change calculated and adjusted to control values. Data are represented as mean ± S.E.M. of percent input (EZH2 inhibitor stimulation; *n* = 6). Statistical significance was calculated by comparing control vs GSK126 or Tazemetostat using Student *t*-test, *P < 0.05, **P < 0.01, ***P < 0.001, ****P < 0.0001. **e** GSK126 and Taz stimulate insulin protein expression in CK19+ve cells derived from juvenile T1D and adult non-diabetic donors. DAPI served as a control for nuclear staining. Images are representative of pharmacological EZH2 inhibition (*n* = 3). Scale bar represents 100 μm. White arrows point to CK19^+^INS^+^ cells. **f** Glucose responsiveness in exocrine tissue was assessed through a 5-step process: Day 1 pancreatic removal and isolation followed by 48-hour stimulation with GSK126 or Tazemetostat. The assay for glucose-stimulated insulin secretion was performed on Day 5, using low and high-glucose Kreb’s buffer. Insulin concentration was determined by ELISA. **g** Fold change of insulin release in low (2.8 mM) and high (28 mM) glucose conditions from GSK126 and Taz stimulated cells in juvenile T1D and adult non-diabetic donors. Data of two replicate experiments represented as mean ± S.E.M of fold change relative to control. Statistical significance was calculated by comparing 2.8 mM vs 28 mM glucose using Student *t*-test, *P < 0.05, **P < 0.01

To determine if default silencing of the β-cell markers, *PDX1* and *INS* in pancreatic exocrine cells is consistent with a model in which EZH2 is coupled with H3K27me3, we performed chromatin immunoprecipitation (ChIP) experiments from diabetic and non-diabetic donor cells stimulated with GSK126 and Taz (Fig. 2a). Chromatin-associated histones were cross-linked in situ followed by sonication. DNA associated with methylated H3K27 was immunoprecipitated using an antibody that specifically recognises trimethylation of histone 3 (H3K27me3). The immunopurified chromatin associated with DNA was assessed by qPCR using amplimers that were specifically designed to detect regions of the *NGN3, PDX1, INS-IGF2, GCK, MAFA, PCSK1*, *PCSK2* and *CK19* genes. EZH2 inhibition with GSK126 and Taz reduced H3K27me3 content on the *PDX1* gene for the juvenile T1D (Fig. 2b) adult T1D (Fig. 2c) and adult non-diabetic (Fig. 2d) donors. Similarly, H3K27me3 associated *INS* DNA levels were reduced from immunoprecipitations using H3K27me3 antibody from exocrine CK19+ve cells stimulated with the small molecule inhibitors. Moreover, we observe reduced H3K27me3 content on *MAFA, GCK, PCSK1* and *PCSK2* genes.

While chromatin immunoprecipitation analyses confirmed the EZH2 inhibitors reduced H3K27me3, GSK126 and Taz did not affect histone acetylation levels at the same lysine site (H3K27ac). Since bivalent domains marked by H3K27me3 and H3K4me3 have been proposed to act on histone patterning of poised genes, we also assessed whether pharmacological inhibition of EZH2 could reverse default suppression by modifying histone bivalency. Chromatin immunoprecipitation analyses revealed elevated H3K4me3 content at *PDX1* and *INS* promoter regions including the regenerative genes implicated in the restoration of β cell-like function. We propose histone bivalency protects reversibly repressed genes from default or irreversible transcriptional suppression. These findings show a close correspondence between default suppression and exocrine regenerative capacity that can be targeted by EZH2 inhibition.

### Ex vivo exocrine cells are capable of expressing insulin

If default suppression by EZH2 is responsible for diminishing exocrine competence by writing H3K27me3 on the *INS* gene, then pharmacological demethylation should also influence the production of INS protein. Diabetic and non-diabetic CK19+ve exocrine cells were stimulated with GSK126 or Taz for 2 days and then monitored using immunofluorescence staining by microscopy (Fig. 2e). EZH2 inhibitors stimulated the production of insulin by immunofluorescence staining in CK19+ve ductal cells at a frequency of 3 insulin positive cells per 20,000 CK19+ve cells, a phenotype not observed in the DMSO controls.

### Exocrine cells derived from juvenile T1D and adult non-diabetic donors are capable of releasing insulin

Having demonstrated that the refractory nature of chromatin influences the expression of *INS* mRNA and protein in exocrine cells, we examined whether EZH2 inhibition could also stimulate insulin secretion. To assess the regenerative capacity of diabetic exocrine cells stimulated with GSK126 and Taz, we developed a glucose-stimulated insulin secretion (GSIS) protocol (Fig. 2f). This assay evaluates ductal cell functionality to produce insulin under basal (2.8 mM glucose) and hyperglycaemic (28 mM glucose) conditions. Stimulation with GSK126 and Taz influenced glucose-responsive insulin secretion in diabetic and non-diabetic exocrine cells (Fig. 2g). These results suggest key metabolic markers of glucose homeostasis and mature β-cell activity are functional.

### Inhibition of EZH2 in human pancreatic ductal epithelial cells promotes β-cell indices

**Fig. 3:**
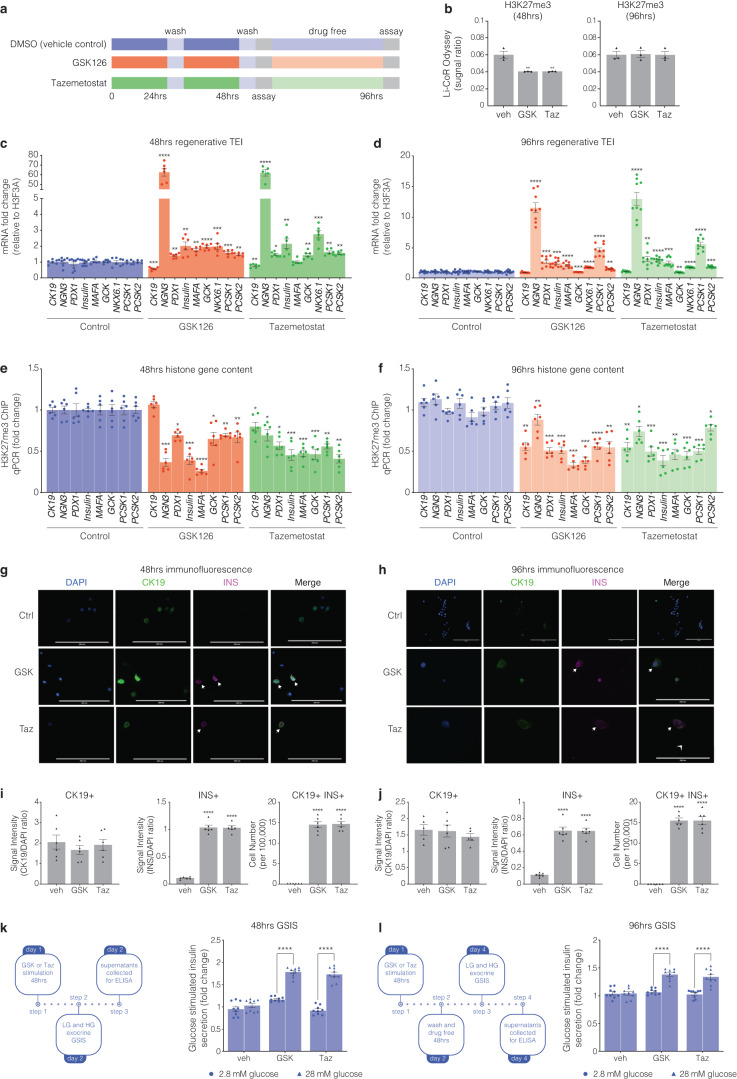
Human pancreatic ductal epithelial cells express β-cell indices in response to pharmacological EZH2 inhibition. **a** Cells were stimulated with GSK126 or Tazemetostat over a 48-hour period. Assays were then performed at the 48-hour time point as well as 48 h following drug free conditions at the 96-hour time point. **b** Histones were prepared by acid extraction. Quantification of H3K27me3 levels were calculated and adjusted to overall histone H3 using Li-COR Odyssey. The signal ratio for H3K27me3/total H3 was calculated at 48 and 96 h. Vehicle control is DMSO. Data are presented as mean with error bars as S.E.M of 3 replicates of stimulation. Statistical significance was calculated by comparing control vs inhibitor values using Student *t*-test, **P < 0.01. **c** Regenerative TEI of *CK19, NGN3, PDX1, INS, MAFA, GCK, NKX6.1, PCSK1*, and *PCSK2* in pancreatic ductal cells after 48 h stimulation with GSK126 or Tazemetostat assessed by qRT-PCR, normalised to *H3F3A* and adjusted to controls. Data are represented as mean of 3 replicates. Statistical significance was calculated by comparing control vs inhibitor values using Student *t*-test, *P < 0.05, **P < 0.01, ***P < 0.001, ****P < 0.0001, error bars are S.E.M. **d** Regenerative TEI of *CK19, NGN3, PDX1, INS, MAFA, GCK, NKX6.1, PCSK1*, and *PCSK2* in pancreatic ductal cells after 48 h stimulation with GSK126 or Tazemetostat followed by 48 h drug free conditions (96 h) assessed by qRT-PCR, normalised to *H3F3A* and adjusted to controls. Data are represented as mean of 3 replicates. Statistical significance was calculated by comparing control vs inhibitor values using Student *t*-test, **P < 0.01, ***P < 0.001, ****P < 0.0001, error bars are S.E.M. **e** Chromatin immunoprecipitation of H3K27me3 content for regenerative genes include *CK19, NGN3, PDX1, INS-IGF2, MAFA, GCK, PCSK1*, and *PCSK2* in pancreatic ductal cells following 48 h of GSK126 or Taz stimulation assessed by qPCR and represented as fold change, normalised and adjusted to controls. Data are represented as mean ± S.E.M. of percent input (GSK126 or Taz stimulation; *n* = 3). Statistical significance was calculated by comparing control vs GSK126 or Taz using Student *t*-test, *P < 0.05, **P < 0.01, ***P < 0.001, ****P < 0.0001. **f** Chromatin immunoprecipitation of H3K27me3 content for regenerative genes include *CK19, NGN3, PDX1, INS-IGF2, MAFA, GCK, PCSK1*, and *PCSK2* in pancreatic ductal cells after 48 h stimulation with GSK126 or Taz followed by 48 h drug free conditions (96 h) assessed by qPCR and represented as fold change, normalised and adjusted to controls. Data are represented as mean ± S.E.M. of percent input (GSK126 or Taz stimulation; *n* = 3). Statistical significance was calculated by comparing control vs GSK126 or Taz using Student *t*-test, *P < 0.05, **P < 0.01, ***P < 0.001, ****P < 0.0001. **g** Immunofluorescence staining of human pancreatic ductal cells stimulated after 48 h stimulation with GSK126 or Taz. Cells were stained for DAPI, CK19, and INS. Images across three replicates of stimulation were captured at 20x magnification using ThermoFisher EVOS and processed with ImageJ. Scale bar represents 200 μm. Arrows point to CK19^+^INS^+^ cells. **h** Immunofluorescence staining of human pancreatic ductal cells stimulated after 48 h stimulation with GSK126 or Taz followed by 48 h drug free conditions (96 h). Cells were stained for DAPI, CK19, and INS. Images across three replicates of stimulation were captured at 20x magnification using ThermoFisher EVOS and processed with ImageJ. Scale bar represents 200 μm. Arrows point to CK19^+^INS^+^ cells. **i** Quantifiction of immunofluorescence staining of human pancreatic ductal cells stimulated after 48 h stimulation with GSK126 or Taz. Protein expression was quantified by normalizing the CK19+, INS+ and CK19+/INS+ signals relative to the nuclear DAPI signal. Insulin expressing cells were scored across a total of 1 × 10^5^ cells seeded on coverslips. Data are represented as mean ± S.E.M. of 6 replicates. Statistical significance was calculated by comparing control vs GSK126 or Taz using Student *t*-test, ****P < 0.0001. **j** Quantifiction of immunofluorescence staining of human pancreatic ductal cells stimulated after 48 h stimulation with GSK126 or Taz followed by 48 h drug free conditions (96 h). Protein expression was quantified by normalizing the CK19+, INS+ and CK19+/INS+ signals relative to the nuclear DAPI signal. Insulin expressing cells were scored across a total of 1 × 10^5^ cells seeded on coverslips. Data are represented as mean ± S.E.M. of 6 replicates. Statistical significance was calculated by comparing control vs GSK126 or Taz using Student *t*-test, ****P < 0.0001. **k** Glucose-stimulated insulin secretion assay assessed the regenerative capacity in human pancreatic ductal cells after 48-hour stimulation with GSK126 or Taz. Cells were exposed to low (2.8 mM) and high (28 mM) glucose conditions. Insulin secretion was quantified by ELISA. Fold changes in insulin release are shown for both glucose conditions. Data are of three replicate experiments represented as mean ± S.E.M of fold change relative to control. Statistical significance was calculated by comparing 2.8 mM vs 28 mM glucose using Student *t*-test, ****P < 0.0001. **l** Glucose-stimulated insulin secretion assay assessed the regenerative capacity in human pancreatic ductal cells after 48-hour stimulation with GSK126 or Taz followed by 48 h drug free conditions (96 h). Cells were exposed to low (2.8 mM) and high (28 mM) glucose conditions. Insulin secretion was quantified by ELISA. Fold changes in insulin release are shown for both glucose conditions. Data are of three replicate experiments represented as mean ± S.E.M of fold change relative to control. Statistical significance was calculated by comparing 2.8 mM vs 28 mM glucose using Student *t*-test, ****P < 0.0001

To control for the purity of the cell population and assess the temporal effects of EZH2 inhibitors, human derived pancreatic ductal epithelial cells were stimulated for 48 h with GSK126 or Taz and returned to drug free media for 48 h (Fig. 3a). Assessment of acid purified histone-binding proteins shows GSK126 and Taz stimulation at 48 h diminishes EZH2 mediated H3K27me3 content when compared to the recovery of overall unmodified histone H3 (Fig. 3b). Furthermore, H3K27me3 content was restored following 48 h of drug free conditions when quantified by Li-CoR Odyssey. Moreover, stimulation of pancreatic ductal cells with GSK126 and Taz for 48 h influenced the transcriptional expression index (TEI) of the endocrine progenitor *NGN3*, along with *INS*, and *PDX1* as well as the glucose sensing *GCK* and insulin processing enzymes *PCSK1* and *PCSK2* (Fig. 3c). We observe modest persistence of the TEI when drug stimulated cells are returned to drug free conditions at 96 h (Fig. 3d). We examined the reversibility of default suppression by determining H3K27me3 content for regenerative genes by ChIP. We show that pancreatic ductal cells stimulated for 48 h with GSK126 or Taz have reduced H3K27me3 gene content (Fig. 3e) which is modestly reduced at 96 h (Fig. 3f) when compared to vehicle treated cells.

To ascertain functional protein synthesis of INS and CK19, drug-stimulated cells were analysed by immunofluorescence staining, with DAPI serving as a control for nuclear staining (Fig. 3g at 48 h and Fig. 3h at 96 h). No correlation was observed for insulin expression and the ductal cell marker CK19 (Fig. 3i). Drug stimulation for 48 h identified on average 15 CK19/INS positive cells per 100,000 human pancreatic ductal cells. Whilst this number went unchanged following the removal of EZH2 inhibitors, we observed hypertrophy of insulin positive cells, which was correlated with a reduction in the insulin signal (Fig. 3j). We established a GSIS protocol to determine insulin secretion in human ductal cells stimulated with GSK126 or Taz for 48 h followed by high glucose exposure (left insert Fig. 3k). Insulin secretion was elevated in human pancreatic ductal epithelial cells stimulated with GSK126 or Taz (right insert Fig. 3k). We also determined insulin secretion following 48 h drug free conditions (96 h, left insert Fig. 3l). While removal of GSK126 and Taz after 48 h lowered insulin secretion, glucose stimulated insulin secretion was higher than the vehicle control (right insert Fig. 3l).

### Human β-cells are characterised by permissive chromatin domains that regulate transcriptional competence

**Fig. 4:**
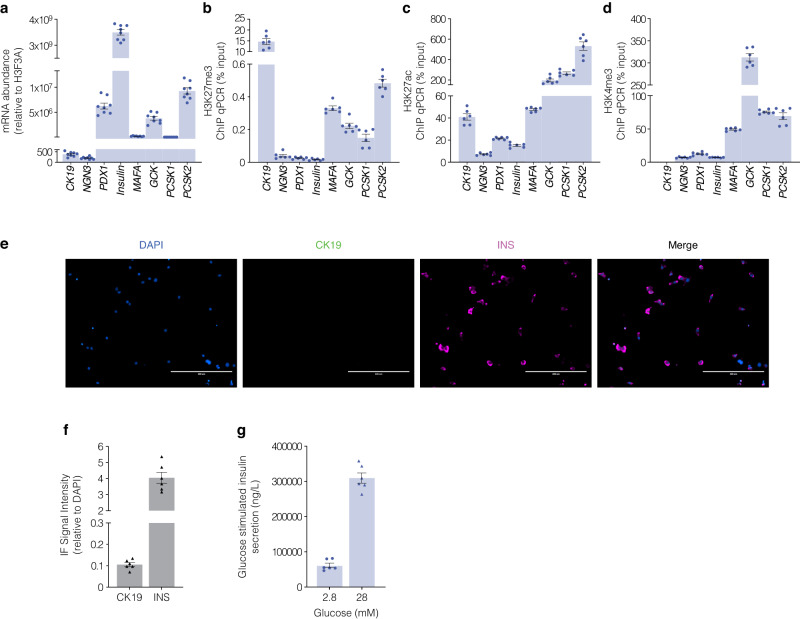
Characteristic transcriptional indices of β-cells show robust insulin signal underscored by reduced H3K27me3 gene content. **a** The expression of mRNA transcripts for *CK19, NGN3, PDX1, INS, MAFA, GCK, PCSK1* and *PCSK2* genes from mature EndoC-βH5 cells relative to *H3F3A* are displayed. Data are represented as mean of 3 replicates, error bars are S.E.M. **b** Chromatin immunoprecipitation for suppressive H3K27me3 chromatin content for *CK19, NGN3, PDX1, INS-IGF2, MAFA, GCK, PCSK1* and *PCSK2* genes derived from EndoC-βH5 cells. Data are represented as mean ± S.E.M. of percent input (*n* = 3). **c** Chromatin immunoprecipitation for permissive H3K27ac chromatin content for *CK19, NGN3, PDX1, INS-IGF2, MAFA, GCK, PCSK1* and *PCSK2* genes derived from EndoC-βH5 cells. Data are represented as mean ± S.E.M. of percent input (*n* = 3). **d** Chromatin immunoprecipitation for permissive H3K4me3 chromatin content for *CK19, NGN3, PDX1, INS-IGF2, MAFA, GCK, PCSK1* and *PCSK2* genes derived from EndoC-βH5 cells. Data are represented as mean ± S.E.M. of percent input (*n* = 3). **e** Immunofluorescence staining confirms functionally mature EndoC-βH5 cells. Staining was performed for DAPI, CK19, and INS. Images across three replicates of stimulation were captured at 20x magnification using ThermoFisher EVOS and processed with ImageJ. Scale bar represents 200 μm. White arrows point to CK19^+^INS^+^ cells. **f** Protein signal of immunofluorescence staining positive for INS in the absence of CK19 in EndoC-βH5 cells. Staining was assessed by adjusting the CK19 or INS signals to the nuclear DAPI signal. Data are represented as mean ± S.E.M. **g** Glucose stimulated insulin assays assessed in EndoC-βH5 cells. Fold change of insulin release in low (2.8 mM) and high (28 mM) glucose conditions. Data are of three replicate experiments represented as mean ± S.E.M of insulin concentration

These studies sugest the refractory nature of chromatin defines exocrine suppression and β-cell plasticity following pancreas surgical resection from juvenile and adult T1D donors. This closely corresponds with transcriptional indices influenced by EZH2 inhibition using human pancreatic ductal epithelial cells. If EZH2 protects reversibly suppressed genes from regenerative silencing in pancreatic ductal cells, then β-cell gene content should be reduced for H3K27me3 and transcriptionally converted to an active state. Baseline transcriptional profiling of EndoC-βH5 human pancreatic β-cells showed characteristic insulin expression and robust expression of the *PDX1*, *MAFA* and *GCK* genes (Fig. 4a). Because insulin is synthesized in pancreatic β-cells from its precursor proinsulin which is cleaved by the prohormone convertases to generate mature insulin we also examined prohormone expression of *PCSK1* and *PCSK2* (Fig. 4a). As anticipated the expression of pancreatic ductal cell marker *CK19* and progenitor marker *NGN3* were barely detectable in mature EndoC-βH5 cells. To determine if the silencing of regenerative capacity in pancreatic ductal cells is inverted in EndoC-βH5 cells we assessed H3K27me3 gene content by chromatin immunoprecipitation (Fig. 4b). DNA associated with hypermethylated H3K27me3 was immunoprecipitated and DNA-bound histones recovered and analysed by qPCR. We recovered robust *CK19* DNA levels from EndoC-βH5 cells consistent with the β-cell phenotype. Moreover, we barely detected H3K27me3 chromatin content for β-cell genes *PDX1* and *INS* including modest enrichment on *MAFA, GCK, PCSK1* and *PCSK2* genes which is consistent with transcriptional competence. Conversely, ChIP analyses of permissive chromatin content influencing transcriptional activation were also examined in EndoC-βH5 cells showing reciprocal association for H3K27ac (Fig. 4c) including H3K4me3 content (Fig. 4d) for the β-cell gene markers. These results show a direct correlation between the loss of H3K27me3 with enhanced H3K27ac and H3K4me3 at genes functionally linked with human β-cell maturity that were also supported by immunofluorescence staining (Fig. 4e) CK19/INS signals (Fig. 4f) and consistent with glucose stimulated insulin release in the EndoC-βH5 cells (Fig. 4g). Taken together, these studies suggest the ability to restore regenerative β-cell capacity from pancreatic ductal cells might be associated with default suppression that can be targeted by pharmacological inhibition of the EZH2 methyltransferase.

## Discussion

Until now, the regenerative process and default suppression have been incidental, lacking confirmation. The rare opportunity to examine fresh tissue resected from a donor and the availability of Tazemetostat, the second EZH2 inhibitor approved by the FDA, has allowed a better characterisation of the refractory nature of chromatin underscoring the regenerative barrier of ductal cells derived from the pancreas in diabetic mellitus. Building upon recent and previous studies, we expand age-independent endocrine reprogramming of exocrine tissue isolated from type 1 diabetics.

β-cell replacement remains an important requirement for the treatment of insulin-dependent diabetes. The rate of transplantations is unmet by donor numbers. Furthermore, approximately 3 pancreata are required to generate sufficient islet equivalents for transplantation into a single recipient.^28^ Therapies that could promote β-cell regeneration could lessen the complications of T1D. However, the epigenetic mechanisms that govern endocrine progenitor regeneration in humans are poorly understood. This gap in knowledge has impeded the development of epigenetic therapies to advance ductal-driven β-cell regeneration. Whilst various strategies to regenerate insulin-producing cells have been previously reported, this study extends the results of a previous study in demonstrating an epigenetic mediated strategy to reprogram terminally differentiated adult human pancreatic ductal cells into insulin-producing β-like cells by specifically addressing default suppression mediated by the EZH2 methyltransferase.^23^

While EZH2 is capable of binding the *NGN3* transcription factor^29^ it is also appreciated the methyltransferase influences endocrine cell fate of *NGN3* positive pancreatic cells.^23^ In this study, gene expression analysis of GSK126 or Taz stimulated cells from a juvenile T1D donor demonstrated elevated expression of the master regulator of pancreatic endocrine cells, *NGN3*, which is transiently expressed and modulates downstream target genes resulting in a transition of cell identity. This is supported by previous studies that have used stem cell differentiation protocols to generate *NGN3* positive cells in mice deleted for EZH2.^30^ Indeed, the transcriptomic analyses of our study identify genes critical for pancreatic function, β-cell development and insulin regulation in the juvenile T1D.

RNA sequencing of cells stimulated with GSK126 and Taz showed elevated expression of the *ISL1, NEUROD1, PTF1A* and *FGF10* genes in juvenile T1D donor that is indicative of coordinated β-cell neogenesis and maturation. Moreover, *FGF10* signalling helps preserve the pancreatic pool of progenitor cells,^31^ while *ISL1, NEUROD1* and *PTF1A* promote the differentiation and maturation of β-cells.^32,33^ The observed increase in *INS* expression is likely a result of the enhanced activity of these transcription factors that are known to influence differentiation and maturation of insulin-producing β-cells. However, our analyses were limited as they are based on a small number of patient-derived tissues and the findings influenced by individual transcriptomes possibly obscure broader trends relevant to β-cell regeneration. Further work will also be required in determining the general applicability of pharmacological EZH2 inhibition in T1D donors with low or high residual β-cell activity.^34,35^

**Fig. 5:**
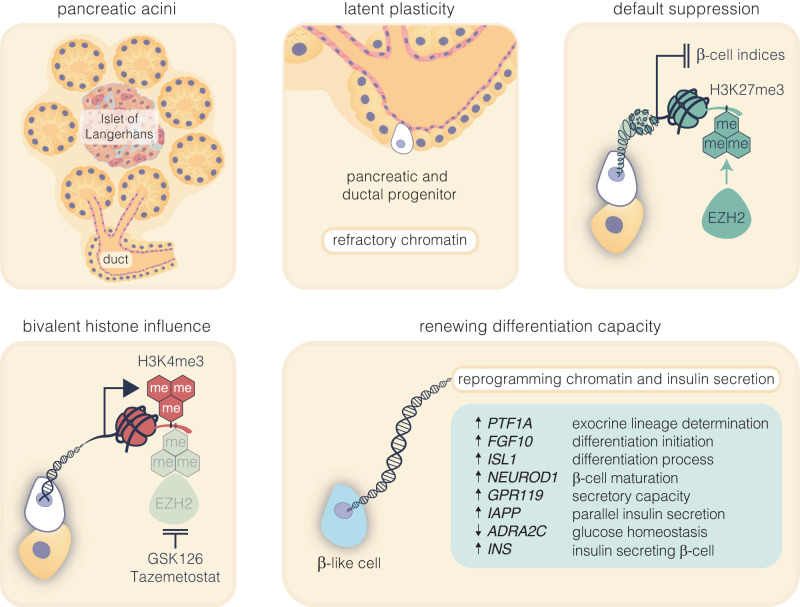
Pharmacological inhibition of EZH2 catalyses pancreatic progenitor activation and β-cell maturation. The schematic outlines the progression from pancreatic multipotent progenitors to mature insulin-secreting β-cells, highlighting the regulatory target of EZH2 inhibitors, GSK126 and Tazemetostat. These progenitors, originating in the Islets of Langerhans’ pancreatic ducts, are maintained in a multipotent state post-development associated with EZH2-mediated suppression with H3K27me3 content enriched on endocrine genes. Reducing H3K27me3 levels shifts bivalent H3K4me3 mark on these progenitors towards the endocrine lineage, marked by *PTF1A* activation and primes these cells for β-cell differentiation. While *FGF10* signalling stabilises this progenitor state, *ISL1* and *NEUROD1* influence endocrine commitment that support β-cell maturation. Upregulation of *GPR119* and *IAPP*, along with the downregulation of *ADRA2C*, weakens inhibitory signals, facilitating glucose-stimulated insulin secretion

The ability to reactivate transcriptional activity of key regenerative genes by EZH2 inhibition in human pancreatic ductal epithelial cells is in accordance with chromatin modification and reduced H3K27me3. Whereas bivalency protects reversibly repressed genes from default silencing, EZH2 inhibition effectively elevated H3K4me3 thereby influencing regenerative competence. For example, the RNA-seq data of donor and ductal epithelial cells exposed to the small molecule inhibitors effectively restored *GPR119* receptor expression and is consistent with its role in glucose-dependent insulin secretion in the pancreas.^36^ Under the same ex vivo conditions we observe robust expression of the *IAPP* gene which closely correspond with metabolic studies demonstrating enhanced insulin secretory response. This elevation in *IAPP*, typically co-secreted with insulin from β-cells^37^ alongside regulation of *PTF1A* and *ADRA2C* molecules suggest the pancreatic ductal cells could be transitioning towards a β-like cell identity. Indeed, increased *PTF1A* expression is suggestive of cellular differentiation,^38^ whereas downregulation of the insulin inhibitor expression, *ADRA2C*^39^ is consistent with its role in improved insulin secretion. Collectively, our findings from human ductal cells not only shine a light on the regulatory mechanisms governing pancreatic function but also underscore the plasticity of ductal cells derived from the pancreas. Although other regulatory factors will need to be considered, including more efficient methods of regeneration, our data reveal details of transcriptional control by targeting EZH2 to adopt a β-like cell phenotype and contribute to insulin secretion (Fig. 5). In addition, while transcriptomic ductal cell data has restricted β-cell transition, an improved expression signature would likely be achieved by specific enrichment and analysis of target cells that are transitioning to a β-cell status.

The upregulation of *PDX1* is crucial for initiating the development of the pancreas, however post-development, the expression of *PDX1* is confined to mature β-cells where it is responsible for maintaining the production of insulin.^40^ In agreement with the previous observations and despite the destruction of β-cells in the islets, we found that stimulation of pancreatic ductal cells with GSK126 and Taz could influence *INS* gene expression and was correlated with the expression of maintenance markers that define the β-cell, namely *PDX1* and *MAFA*. Furthermore, we demonstrate for the first time that Taz could restore *INS* expression reinforcing the functional importance of chromatin content regulating transcriptional suppression. Despite structural differences of the two compounds, molecular dynamics simulations show SET domain inhibition that is consistent with previous studies of the human methyltransferase.^24^ While GSK126 and Taz are competitive EZH2 inhibitors, their influence on gene expression were distinct yet comparable. Whether this reflects differences in dosing schedules is unclear. Our studies with Taz confirm that EZH2 limits human exocrine regenerative capacity, while enzyme inhibition offers a possible strategy to influence regeneration without significantly affecting cell viability (Supplementary Fig. 1). In addition, our ability to procure tissues from a non-diabetic donor including juvenile T1D donor (7 yrs of age and 1 month diabetes duration) and adult T1D donor (61 yrs of age and 33 yrs diabetes duration) has allowed us to support the notion of regenerative capacity beyond the original single-case study^23^ (donor characteristics are summarised in Supplementary Table 1). The regenerative β-cell-like signature was also observed in human pancreatic ductal epithelial cells and conversely associated with studies using functionally mature EndoC-βH5 human β-cells.

The results presented are consistent with previous studies investigating the histone modification status of pancreatic endocrine genes^41^ with a summary of β-like cell indices shown in Supplementary Table 2. We have shown endocrine and β-cell genes such as *PDX1* and *MAFA* are bivalently primed by H3K4me3 with parallel loss of the repressive H3K27me3 thereby influencing ductal progenitor identity. Furthermore, the expression of the ductal cell marker, *CK19* was reduced in T1D exocrine cells stimulated with Taz alongside expression levels of the insulin processing enzymes *PCSK1* and *PCSK2*. Taken together, these results are suggestive of ductal cell transition.

There was a corresponding increase in gene expression of mature insulin. This correlation was evident at the protein level in a population of cells as shown by immunofluorescence. This was observed in the non-diabetic and T1D donors including pancreatic ductal epithelial cells. This is correlated with a 20% increase of insulin protein secreted into media under glucose conditions when cells were stimulated with GSK126 or Taz. The outcomes of the GSIS assay underscore a significant characteristic of the differentiated insulin-secreting cells. Indeed, these studies suggest for the first time the ability of β-like cells to respond to dynamic changes in glucose levels. It is important to note that for transitioning β-cells, enhanced insulin production and secretion observed is in the picomolar range and in contrast to functionally mature β-cells.^40^ This is likely to be associated with the number of insulin secreting cells when compared to overall exocrine fraction, as determined by immunofluorescence imaging.

These studies also show temporal regulation of gene expression in pancreatic ductal epithelial cells with GSK126 and Taz. We observe significant upregulation of β-cell indices following transient 48 h exposure with the drugs and the concomitant reduction in H3K27me3 modification on genes which closely corresponds with elevated H3K4me3. We propose bivalent trimethylation marks at lysine positions 4 and 27 of histone H3 protects transcriptionally suppressed genes from irreversible silencing. This is consistent with restored H3K4me3 gene content and is likely to influence β-cell capacity. The inability to persistently reactivate transcription in drug free conditions following removal of GSK126 and Taz is supported by the reversibility of overall H3K27me3 content in pancreatic cells. Closer examination of transcriptional expression indices lends further support to regenerative reversibility. Indeed, despite drug free conditions at 96 h, robust transcriptional output remains and closely corresponds with reduced H3K27me3 gene content. The therapeutic implications of these findings for β-cell regeneration are clearly complicated. First, the human data supports targeting EZH2 to influence regenerative indices associated with β-cell development. However, the reversibility of the effects upon drug removal highlights the importance of sustained modulation of the epigenetic landscape to achieve long-lasting therapeutic outcomes. Moreover, it underscores the need for further studies to understand the temporal dynamics of EZH2 inhibition and its potential impact on β-cell differentiation.

We propose a pivotal epigenetic shift promotes the differentiation trajectory of pancreatic ductal progenitor cells towards a β-cell identity. Indeed, our investigation of regenerative transcriptional indices characterising pancreatic ductal cells with functionally mature EndoC-βH5 human β-cells emphasise this paradigm (Supplementary Fig. 2a). A particularly striking observation was the non-refractory status of regenerative H3K27me3 gene content observed in mature EndoC-βH5 cells contrasts with H3K27me3 gene content observed in naïve human pancreatic ductal epithelial cells (Supplementary Fig. 2b). The diminished H3K27me3 gene content observed in EndoC-βH5 cells serves as an indicative epigenetic signature suggesting trajectorial progression towards a functionally mature β-cell phenotype. This was also supported by protein expression using immunofluorescence showing robust insulin staining indicative of β-cell identity. While mature EndoC-βH5 cells lacked CK19 expression, under the same experimental conditions our differentiated β-like cells stained positive for insulin and CK19. These studies suggest that despite adopting β-like traits, these cells retain markers of ductal origin, highlighting the transitional state of their differentiation, with lower insulin signals. Indeed, mature EndoC-βH5 cells respond to hyperglycaemia, with a pronounced increase in insulin secretion. This is a fundamental characteristic of functional β-cells. The metabolic capacity observed using ex vivo donor tissue coupled with studies examining human pancreatic ductal cells stimulated with EZH2 inhibitors, reaffirms their β-like cell identity. This not only reinforces the validity of our differentiation protocol but also paves the way for further studies into the epigenetic landscape governing β-cell maturation.

There are study limitations. First, this is only the second case study of progenitor capacity reinstated despite β-cell destruction in a child with T1D, but we are confident that default suppression is part of the reversible silencing process in adult T1D. This study is also the first example of Taz influencing insulin gene and protein expression from exocrine ductal cell fractions derived from the diabetic pancreas. Second and perhaps more important, not all ductal cells are destined to undergo β-cell transition. The inability to convert all ductal cells is in accordance with recent reports that pancreatic *Ngn3* positive progenitors are rare endocrine cells. A recent scRNA-seq survey of human pancreatic cells confirmed a single *Ngn3* positive progenitor from a population of 11,174 postnatal cells.^42^ Based on these estimates we hypothesise the conversion efficiency can be further improved following surgical resection of ductal cells from the pancreas. In any case, the definitive Ricordi/Edmonton technique verified the exocrine pellet containing purified ductal derivatives were metabolically active.^43^ While chromatin coupled regulation of differentiation was confirmed, we cannot rule out sub-optimal conditions used in-situ did not parallel the exact nature of the pancreatic exocrine milieu. An undefined fraction of responsive ductal progenitors remains possible. Nevertheless, it is likely that this second case report of T1D including non-diabetic donors and the detailed studies outlined will open a window to examine the refractory nature of chromatin mediated H3K27me3 silencing which can be reversibly targeted to restore transcriptionally permissive H3K4me3 gene content and β-cell differentiation.

## Conclusion

In diabetes, the most direct mechanism for transcriptional repression independent of DNA sequence is by histone methylation. The death of an insulin-dependent child managed by constant round-the-clock injections for almost four years, is a devastating reminder of the dominant role of default suppression and emphasises its influence on the regenerative barrier.^23^ We hypothesise the inability to reactivate transcription is responsible for suppressing β-cell indices. Combined with the previous study, it is plausible that targeting treatment refractory chromatin may influence regenerative competence. Whilst other factors involved in regulating progenitor expansion will need to be considered, we propose that the specialised chromatin structures assembled for methylation-dependent silencing will need to be overcome to restore or revert precursor capacity. This reawakening might be achieved in resistant exocrine cells by inhibiting EZH2 dependent silencing to regain the ability of β-like cells to produce insulin.

## Materials and methods

### Human samples

We obtained cadaveric pancreatic tissues with informed consent for research purposes from heart-beating, brain-dead donors through the National Islet Transplantation Programme at Westmead Hospital in Sydney and the St. Vincent’s Institute in Melbourne, Australia. These tissues were collected from individuals both with and without diabetes and were processed to isolate islet, acinar, and ductal tissues. Our research received institutional approval from the Human Research Ethics Committee at St. Vincent’s Hospital in Melbourne, under HREC Protocol number: 011/04.

### Preparation and culture of isolated human pancreatic cells

Exocrine tissue was isolated as a by-product of islet isolation by intraductal perfusion and digestion of the pancreas with collagenase AF-1,^44^ (SERVA/Nordmark, Germany), followed by purification using Ficoll density gradients.^45^ The acinar and ductal tissue obtained from high-density fractions were cultured in Miami Media 1 A (Mediatech/Corning 98–021, USA) supplemented with 2.5% human serum albumin (Australian Red Cross, Melbourne, VIC, Australia), in a 37 °C, 5% CO_2_ incubator.

### Ex vivo stimulation of human pancreatic progenitors with GSK126 and Tazemetostat

Human pancreatic exocrine cells were either left untreated or exposed to 10 μM GSK126 (S7061, SelleckChem) or 1 μM Tazemetostat (S7128, SelleckChem) at a density of 1 × 106 cells per well for 24 h. After the initial 24 h incubation, fresh Miami Media was added, and the cells were cultured for an additional 24 h with either 10 μΜ GSK126 or 1 μM Tazemetostat. All incubations occurred in Miami Media 1 A (Mediatech/Corning 98-021, USA) supplemented with 2.5% human serum albumin (Australian Red Cross, Melbourne, VIC, Australia) in a cell culture incubator at 37 °C with 5% CO_2_ for a total of 48 h, using non-treated six-well culture plates (Corning). Because of low cell numbers isolated from the adult T1D donor, harvests were prioritised for gene expression and ChIP analyses, thus data for immunofluorescent staining and GSIS have not been provided.

### Molecular modelling of GSK126 and Tazemetostat bound to EZH2

Molecular dynamic simulations were performed for the PRC2 complex with GSK126 and Tazemetostat bound to the SET domain of EZH2 in triplicate for 200 ns with a time-step of 2 fs using GROMACS 2018.2 and the CHARMM36 force field.^46,47^ Binding free energy of the ligands was calculated on the final 20 ns of trajectories at 10 ps snapshots and decomposed on a per-residue basis using molecular mechanics Poisson-Boltzmann surface area (MM-PBSA).^48^

### RNA isolation and mRNA-seq

Stimulated human ex vivo pancreatic cells and human pancreatic ductal epithelial cells were isolated using TRIzol (Invitrogen), and Total RNA was extracted from the cells using RNeasy Kit (QIAGEN) including a DNase treatment. Bulk RNA-seq sequence reads underwent quality and adaptor trimming with fastp (v0.20.0). Trimmed reads were mapped to human genome build 38 (hg38) using STAR aligner (v2.7.9a) and sorted with samtools (v1.9) before counting of mapped reads against Ensembl gene level annotations (GRCh38.104) using FeatureCounts (subread/2.0.1) to generate a raw gene-sample count matrix. Gene-sample count matrix counts were normalised and analysed using edgeR (v3.42.4) to generate differential gene expression.

### Gene expression analysis

Stimulated human ex vivo pancreatic cells and human pancreatic ductal epithelial cells were isolated using TRIzol (Invitrogen), and Total RNA was extracted from the cells using RNeasy Kit (QIAGEN) including a DNase treatment. A high-capacity cDNA Reverse Transcription Kit (Applied Biosystems) was used on a total of 1000 ng of RNA to perform first-strand cDNA synthesis according to the manufacturer’s instructions. The oligoperfect designer (Thermo Fisher Scientific) was used to obtain primers against specific genes, as shown in Table 1. Quantitative real time PCR analyses were performed with the PrecisionFast 2× qPCR Master Mix (Primerdesign) and primers using Applied Biosystems 7500 Fast Real-Time PCR System. The mix for each qPCR reaction comprised: 5 μl qPCR Master Mix, 0.5 μl of forward and reverse primers, 2 μl nuclease-free water, and 2 μl of the pre-synthesized cDNA, diluted 1/20. Expression levels of specific genes were tested using the 2-^ΔΔCT^ method with test CT values normalised to *H3F3A* housekeeping gene.

**Table 1:** Human cDNA primers for Real Time Quantitative PCR Assays

Gene	Sequence
*H3F3A*	Forward: ACAAAAGCCGCTCGCAAGAGTG
	Reverse: TTTCTCGCACCAGACGCTGGAA
*INS*	Forward: GCAGCCTTTGTGAACCAACAC
	Reverse: CCCCGCACACTAGGTAGAGA
*NGN3*	Forward: CTAAGAGCGAGTTGGCACTGA
	Reverse: GAGGTTGTGCATTCGATTGCG
*PDX1*	Forward: GAAGTCTACCAAAGCTCACGCG
	Reverse: GGAACTCCTTCTCCAGCTCTAG
*NKX6.1*	Forward: CCTATTCGTTGGGGATGACAGAG
	Reverse: TCTGTCTCCGAGTCCTGCTTCT
*CK19*	Forward: AGCTAGAGGTGAAGATCCGCGA
	Reverse: GCAGGACAATCCTGGAGTTCTC
*GCK*	Forward: CCTGGGTGGCACTAACTTCAG
	Reverse: TAGTCGAAGAGCATCTCAGCA
*MAFA*	Forward: GCTTCAGCAAGGAGGAGGTCAT
	Reverse: TCTGGAGTTGGCACTTCTCGCT
*PCSK1*	Forward: AGCTGGACCTTCATGTGATACC
	Reverse: GCTAGCCTCTGGATCATAGTTGG
*PCSK2*	Forward: GCAACGACCCCTATCCTTACC
	Reverse: TGCAACCTTGGAGTTGTATGC

### Chromatin immunoprecipitation

Assessment of chromatin status in human exocrine and pancreatic ductal epithelial cells using immunoprecipitation assays were performed as previously described.^49,50^ Following stimulation, 1% formaldehyde was used to fix cells for 10 min, followed by quenching of the reaction with 0.125 M glycine for 10 min. Fixed cells were resuspended in sodium dodecyl (lauryl) sulfate (SDS) lysis buffer (1% SDS, 10 mM EDTA, 50 mM Tris-HCl pH 8.1) including a protease inhibitor cocktail (Roche Diagnostics GmBH, Mannheim, Germany) and homogenised followed by incubation on ice for 5 min. Next, sonication was performed on all samples to obtain fragments of 200–600 bp. The chromatin was then resuspended in ChIP Dilution Buffer (0.01% SDS, 1.1% Triton X-100, 1.2 mM EDTA, 16.7 mM Tris-HCl pH 8.0, and 167 mM NaCl) and 20 µl of Dynabeads® Protein A (Invitrogen, Carlsbad, CA, USA) was added and pre-cleared. H3K27me3 (Millipore, Cat# 07-449), H3K27ac (Abcam, Cat# ab4729), and H3K4me3 (Abcam, Cat# ab8580) antibodies were used for immunoprecipitation of chromatin and incubated from 4 h to overnight at 4 °C depending on the antibody used as previously described.^23^ DNA that was immunoprecipitated was collected using magnetic separation, sequentially washed with high and low salt buffers, followed by lithium chloride and TE. DNA was then eluted in a solution of 0.1 M sodium bicarbonate with 1% SDS. To reverse the protein-DNA cross-links, Proteinase K (Sigma, St. Louis, MO, USA) was added, and the mixture was incubated at 62 °C for two hours. H3K27me3, H3K27ac, and H3K4me3 enriched DNA was purified using Nucleospin columns (Machrey-Nagel GmbH&Co, Germany). ChIP primers (shown in Table 2) were designed using the integrative ENCODE resource,^51^ and used to assess changes in levels of DNA associated with H3K27me3, H3K27ac, and H3K4me3.

**Table 2:** Human primers for ChIP q-PCR

Gene	Primer	Sequence
*INS-IGF2*	PromR1 Forward	GGGAACATAGAGAAAGAGGTCTCA
	PromR1 Reverse	AATTAATCTCAGCTTCCCCCTAAC
*PDX1*	PromR1 Forward	TGGCTGTGAACAAACTTCATAAAT
	PromR1 Reverse	CACCGTGGCTTAAAAGTTTCTATT
*NGN3*	PromR1 Forward	CTTCTGGTCGCCAAGTTCAG
	PromR1 Reverse	AGCAGATAAAGCGTGCCAAG
*CK19*	PromR1 Forward	GATTCTACAGAACCCCAGCACTAT
	PromR1 Reverse	GAAATAGGTATCCTCCTCCTCCTC
*GCK*	PromR1 Forward	CCCATTATCTGCAATGGCCC
	PromR1 Reverse	TGGACGAGAGCTCTGCAAAC
*MAFA*	PromR1 Forward	CTCCGAAAACGGGCAGATCC
	PromR1 Reverse	CTCTTTGGACTAGCCGGGAG
*PCSK1*	PromR1 Forward	TCTCCGCTGCCCATTCATTG
	PromR1 Reverse	GCGAGTGTGTGAGCTATGGA
*PCSK2*	PromR1 Forward	TAACTTAGTTGCCCTGCCC
	PromR1 Reverse	TAGTTGGGGAACGCATAGCC

### Culture and treatment of immortalised human pancreatic ductal epithelial cells

Immortalized human derived pancreatic ductal epithelial cells of normal phenotype and genotype were obtained (AddexBio, Cat#:T008001) and maintained following the manufacturer’s instructions. In brief, cells were cultures at 37 °C in an atmosphere of 5% CO_2_ in complete Keratinocyte Serum-Free Media (supplemented with human recombinant EGF, Bovine Pituitary Extract and Antibiotic-Antimycotic [Gibco]). Cells were stimulated with EZH2 inhibitors using the same protocol for ex vivo human exocrine tissue. In brief, cells were seeded on Day 0 and allowed to adhere for 24 h. Stimulation was initiated on Day 1 with the first dose of GSK126 or Tazemetostat. Following a further 24 h of culture, the second dose was delivered in fresh K-SFM for a total of 10 μΜ GSK126 or 1 μM Tazemetostat. Cells were then harvested following a total of 48 h incubation in EZH2 inhibitor or DMSO vehicle control. To investigate transient effects of GSK126 and Tazemetostat, cells were cultured in drug free conditions for additional 48 h following which cultures were washed with K-SFM. Cells were incubated for a further 2 days, for a total of 96 h before harvesting.

### Culture of immortalised and functionally mature human β-cells

The EndoC- βH5 cell line was purchased from Human Cell Design and cultured according to the manufacturer’s protocol using the provided reagents. In brief, cells were seeded on plates pre-coated in βCOAT diluted in DMEM (ThermoFisher, #11965092) with 100x penicillin/streptomycin (Gibco). Seeded cells βH5 were maintained at 37 °C in an atmosphere of 5% CO_2_ in the provided ULTIβ medium, with changes performed every 3 days.

### Protein blotting

1 × 10^6^ human pancreatic ductal epithelial cells were seeded in treated 6-well plates (Corning) and stimulated with either GSK126 or Taz as detailed above. Histone proteins were examined as previously described,^52^ using immunoblotting to assess acid purified nuclear proteins. In brief, protein concentrations were determined by incubating samples and standard concentrations of Bovine Serum Albumin (Invitrogen) with Bradford’s Reagent (Sigma). 2 ug of nuclear protein per sample was run on a 4–12% gel (Nu-Page, Invitrogen). Transfer was performed for 2 h using a PVDF membrane (Immobilon-FL; Millipore). Membranes were incubated in primary antibody against H3K27me3 (07-449, Millipore) or H3 (1B1B2, CST) at 4 °C overnight (dilutions listed in Table 2). Membranes were then washed and incubated at r.t.p. for 1 h in fluorescent secondary antibodies against mouse and rabbit (dilutions listed in Table 2). Images of the membranes were obtained using the LiCoR Odyssey infrared system. Quantification of the protein bands was performed using Image Studio with total H3 serving as a loading control.

### Immunofluorescent analysis of ex vivo human exocrine tissue

1 × 10^6^ cells from the donor pancreata were stimulated with EZH2 inhibitors or vehicle control for 48 h. Cells were resuspended in 10% FBS diluted in PBS and 0.1 × 10^6^ cells were spun onto slides and fixed using 4% paraformaldehyde. Permeabilization was performed using 0.1% Triton X diluted in PBS for 10 min, followed by blocking using PBG (0.2% w/v gelatin, 2.5% w/v bovine serum albumin in PBS) for 1 h. Cells were co-stained for CK19 (HPA002465 Sigma-Aldrich), and INS (A0564, DAKO) by incubating overnight at 4 °C using human specific primary antibodies diluted in PBG (dilutions listed in Table 3). Fluorescent secondary antibodies against rabbit (Alexa Fluor 488), and guinea pig (IRDye® 680CW) (dilutions in Table 3) were incubated for 1 h at room temperature. Slides were then incubated with 4′,6-diamidino-2-phenylindole (DAPI) (0.10 μg/ml; D8417 Sigma-Aldrich) for 10 min and coverslips were mounted using Prolong Gold Anti-Fade mountant with DAPI (ThermoFisher). Images were obtained using the EVOS (ThermoFisher) with the TagBFP, Cy5, and GFP filters. Processing and analysis of images was performed using the Image J software.

**Table 3:** Antibody dilutions for western blot and immunofluorescent staining of human T1D donor pancreatic exocrine cells

Antibody	Dilution
Rabbit Anti-CK19	1:200
Guinea Pig Anti-Insulin	1:250
Alexa Fluor 488 Donkey Anti-Rabbit	1:1000
IRDye® 680CW Donkey Anti-Guinea Pig	1:1000
Rabbit Anti-H3K27me3	1:2000
Mouse Anti-Total H3	1:1000
IRDye® 800CW Donkey Anti-Rabbit	1:10000
IRDye® 680CW Donkey Anti-Mouse	1:10000

### Immunofluorescence analyses

0.5 × 10^5^ human pancreatic ductal epithelial cells were seeded on coverslips and stimulated as previously described,^53^ prior to immunofluorescence. In brief, 4% paraformaldehyde was used to fix cells following stimulation with EZH2 inhibitors. Permeabilization was performed using 0.1% Triton X diluted in PBS for 10 min, followed by blocking for 1 h using PBG. Cells were co-stained for CK19 (HPA002465 Sigma-Aldrich), and INS (A0564, DAKO) by incubating overnight at 4 °C using human specific primary antibodies diluted in PBG (dilutions listed in Table 3). Fluorescent secondary antibodies against rabbit (Alexa Fluor 488), and guinea pig (IRDye® 680CW) (dilutions in Table 3) were incubated for 1 h at room temperature. Coverslips were rinsed in PBG and mounted using Prolong Gold Anti-Fade mountant with DAPI (ThermoFisher). Images were obtained with the EVOS (ThermoFisher). Images were processed and analysed using Image J.

### Glucose stimulated insulin secretion assay

1 × 10^6^ cells were seeded in 6-well plates. Cells were stimulated with 10 μM GSK126 and 1 μM Tazemetostat or vehicle control (DMSO) for 48 h in Miami medium and counted using the Cell Countess II to ensure equal number of cells (1 × 10^6^) were used for the GSIS assay. All subsequent washes and incubations were using Krebs Buffer solution (KRB) made using 25 mM HEPES, 115 mM sodium chloride, 24 mM sodium hydrogen carbonate, 5 mM potassium chloride, 1 mM magnesium chloride heptahydrate, 0.1% bovine serum albumin, and 2.5 mM calcium chloride dihydrate dissolved in deionized water and sterile filtered. Following stimulation, the insulin containing Miami medium was removed and cells underwent two washes with 2.8 mM glucose KRB to reduce the background insulin signal. The low glucose insulin secretion was obtained by incubation in 2.8 mM glucose KRB for 1 h. Cells were then cultured in high (28 mM) glucose Krebs Buffer solution for 1 h to obtain the glucose stimulated insulin secretion. The concentration of insulin secreted into the supernatant was assessed using the Ultrasensitive Insulin ELISA (Mercodia) according to manufacturer’s guidelines. The insulin release by cells in response to hyperglycaemia was calculated as a fold change by adjusting inhibitor stimulated concentrations to control concentrations.

### Non-sequential glucose stimulated insulin secretion assay

As recommended by the manufacturer’s protocol, a non-sequential GSIS was performed using mature EndoC-βH5 cells to assay insulin secretion. In brief, 1 × 10^5^ cells were seeded in 96-well plates and cultured for 6 days in UTIβ1 medium provided. The medium was then changed to the starvation medium ULTI-ST the day before the GSIS was carried out. Next, the cells were washed with 0 mM glucose KRB followed by incubation in 2.8 mM or 28 mM glucose KRB for 1 h to obtain the glucose stimulated insulin secretion. The concentration of insulin secreted into the supernatant was assessed using the Ultrasensitive Insulin ELISA (Mercodia) according to manufacturer’s guidelines. The insulin release by cells in response to hyperglycaemia was calculated as a fold change by adjusting inhibitor stimulated concentrations to control concentrations.

### Supplementary information

Supplemental Material and Data

## Data Availability

All data generated and analysed that support the findings of this study are available from the corresponding author upon reasonable request. Sequencing data is available from NCBI’s Gene Expression Omnibus under accession number GSE247275.
