# Supplementary material for: EZH2 inhibitors promote β-like cell regeneration in young and adult type 1 diabetes donors
Source: Signal Transduct Target Ther. 2024 Jan 1;9:2. doi: 10.1038/s41392-023-01707-x (PMC10757994; doi:10.1038/s41392-023-01707-x)
Supplement: Supplementary file 1 — Supplemental Material and Data [file 41392_2023_1707_MOESM1_ESM.docx]

Supplementary Materials for

EZH2 Inhibitors Promote β-like Cell Regeneration in Young and

Adult T1D Donors

EZH2 Inhibitors Advance β-like Cell Regeneration

Keith Al-Hasani, Safiya Naina Marikar, Harikrishnan Kaipananickal, Scott Maxwell, Jun Okabe, Ishant Khurana, Thomas Karagiannis, Julia J. Liang, Lina Mariana, Thomas Loudovaris, Thomas Kay, Assam El-Osta

Correspondence to: [sam.el-osta@baker.edu.au](mailto:sam.el-osta@baker.edu.au)

**This PDF file includes:**

Materials and Methods pg 2

Figures. S1 to S2 pg 3-4

Tables S1 to S2 pg 5-6

**Materials and Methods**

Casapase 3/7 analysis of human pancreatic ductal epithelial cells

2x10^4^ cells/well were seeded in 96-well plates and stimulated with GSK126 or Taz. Caspase concentration was determined using the Apo-ONE® Homogeneous Caspase-3/7 Assay (Promega) following manufacturer’s instructions. In brief, following stimulation, cells were incubated with fresh K-SFM for one hour prior to incubation with Apo-ONE® Caspase-3/7 Assay Reagent and a further incubation at room temperature for 3 hours. Fluorescence was measured using CLARIOstar (BMG Labtech) plate reader (485_Ex_/527_Em_).**
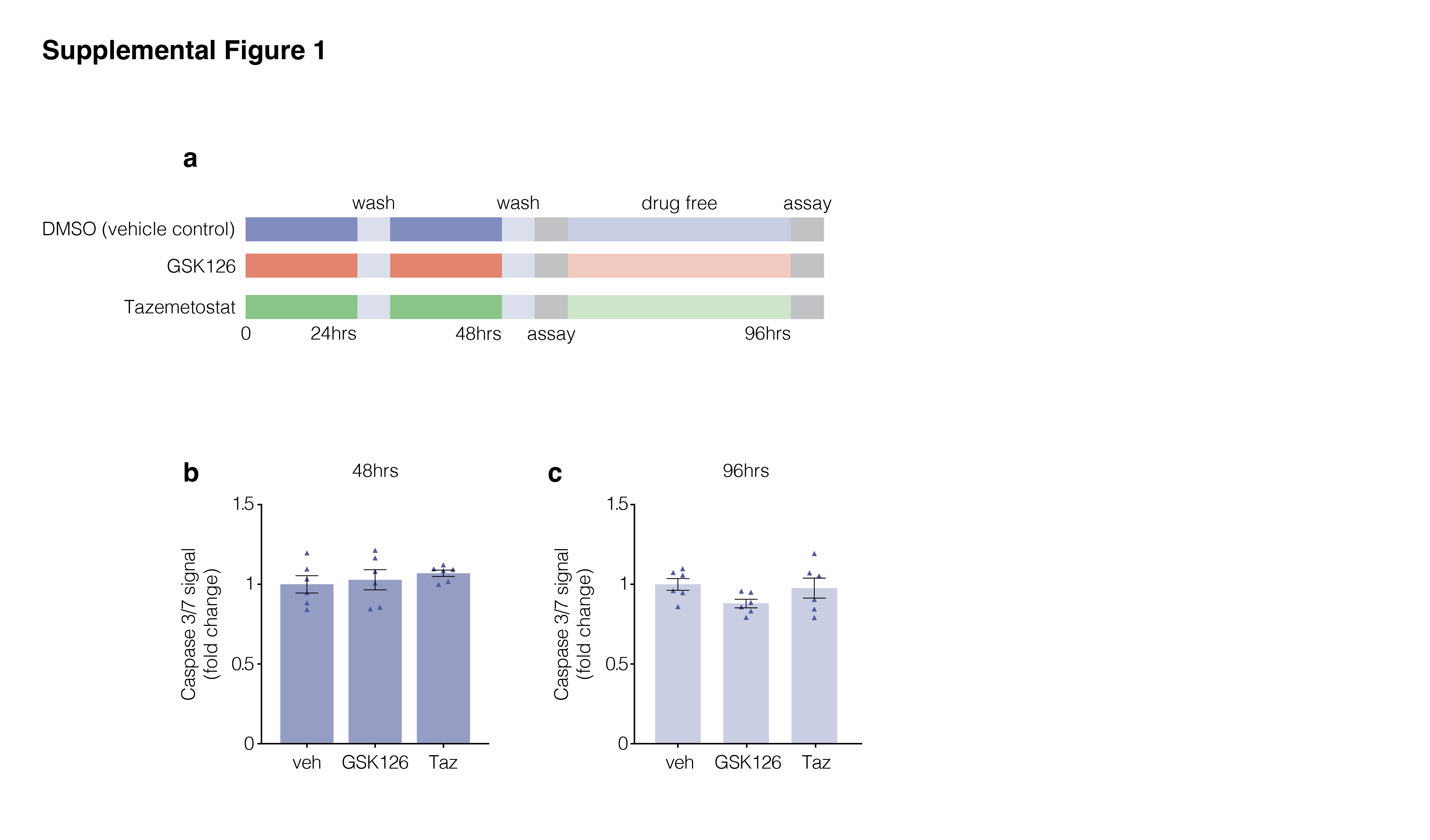
**

**Supplemental Figure 1**.

**a**. Human pancreatic ductal epithelial cells were stimulated with GSK126 or Taz for 48 hours before assessing caspase concentration or allowed to recover for an additional 48 hours in drug free conditions before caspase concentration was determined at 96hrs.

**b**. Caspase activity in cells stimulated with EZH2 inhibitors for 48 hrs. Data are presented as mean with error bars as S.E.M of 6 replicates.

**c**. Caspase activity in cells stimulated with EZH2 inhibitors for 48 hrs and additional 48hr drug free media (96hrs). Data are presented as mean with error bars as S.E.M of 6 replicates.

**
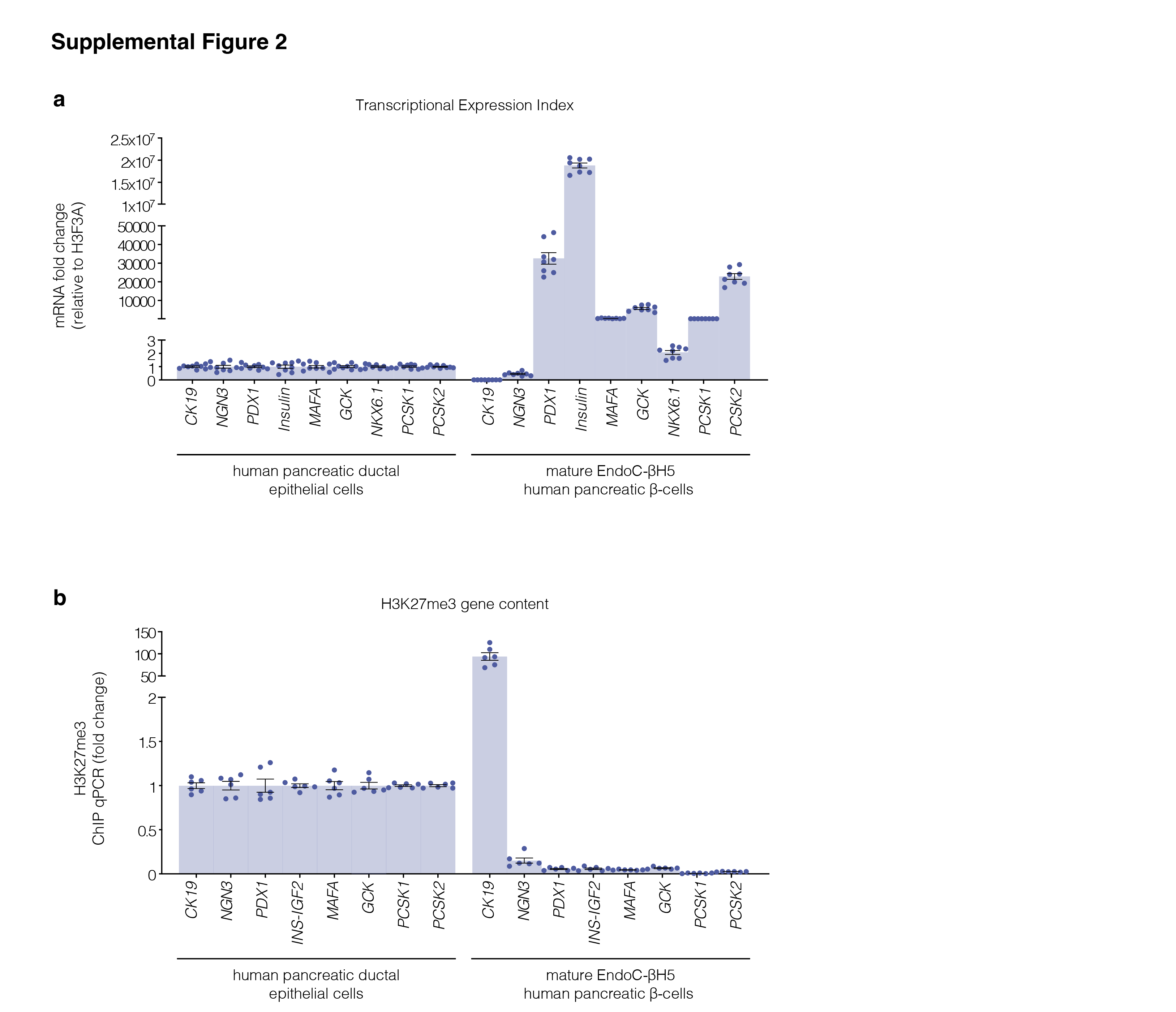
**

**Supplemental Figure 2**.

**a**. Transcriptional expression index of *CK19, NGN3, PDX1, INS, MAFA, GCK, NKX6.1, PCSK1*, and *PCSK2* genes in pancreatic ductal epithelial cells and mature EndoC-βH5 cells are displayed as fold change relative to *H3F3A* calculated and adjusted to ductal epithelial values. Data are represented as mean of 3 experiments with points representing technical replicates, error bars are S.E.M.

**b**. H3K27me3 content for *CK19, NGN3, PDX1, INS-IGF2, MAFA, GCK, NKX6.1, PCSK1,* and *PCSK2* genes in pancreatic ductal epithelial cells and mature EndoC-βH5 cells are displayed as fold change of percent input calculated and adjusted to ductal epithelial values. Data are represented as mean ± S.E.M. of percent input (n=3).

**Supplemental Table 1**. Human donor characteristics.

|  | **Donor 1** | **Donor 2** | **Donor 3** |
| --- | --- | --- | --- |
| Age | 7 | 61 | 56 |
| Sex | M | M | F |
| BMI | 14.1 | 29.3 | 27.0 |
| Diabetes | Type 1 | Type 1 | non-diabetic |
| Diabetes duration | 1 month | 33 years | N/A |
| HbA1c | N/A | 7.1 | N/A |

BMI: Body Mass Index, HbA1c: Hemoglobin A1c, N/A: not available

**Supplemental Table 2**. Regeneration of β-cell indices in non-diabetic and type 1 diabetic human exocrine donor tissue


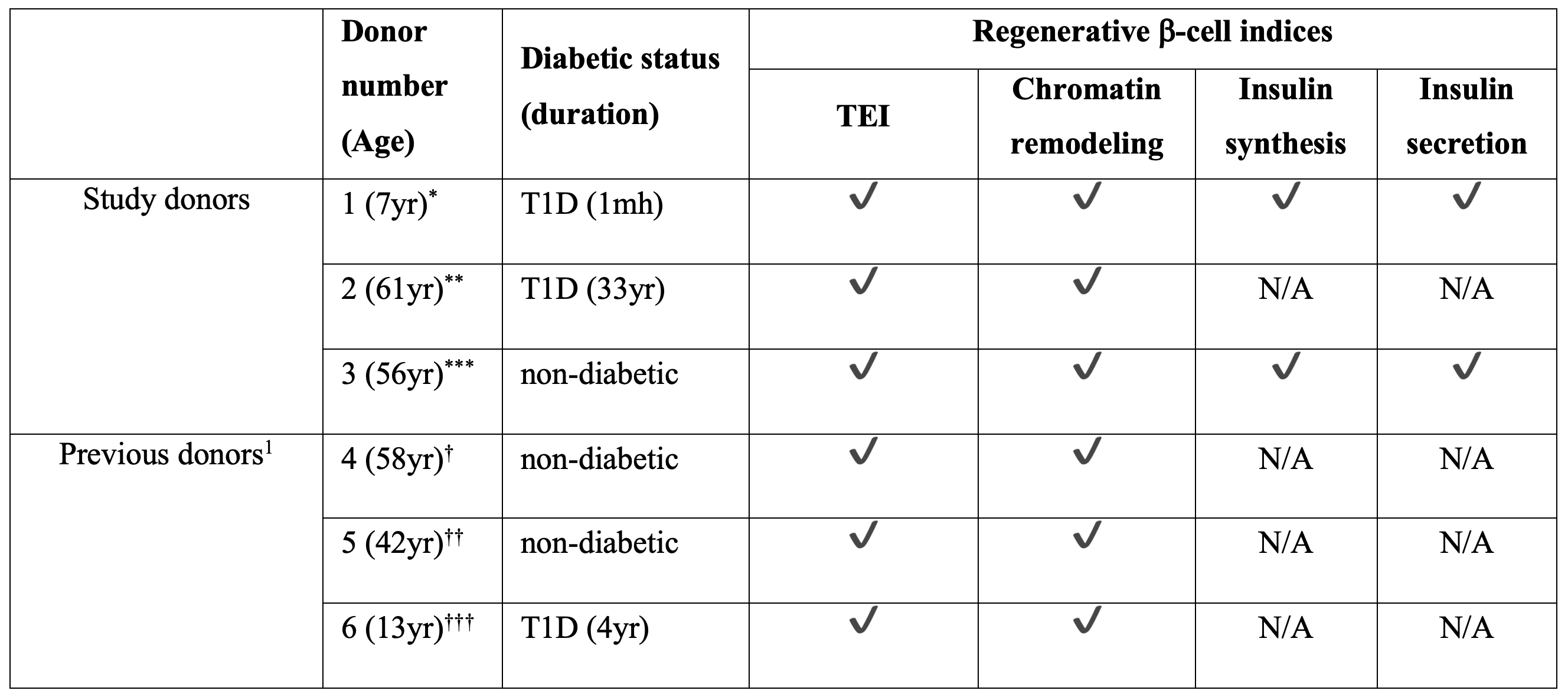


T1D Type 1 Diabetes, N/A not available, TEI Transcriptional Expression Indices, *Donor 1 in Supplementary Table S1, **Donor 2 in Supplementary Table S1, ***Donor 3 in Supplementary Table S1. †Donor 4, ††Donor 5, †††Donor 6 were previously studied, please refer to *Signal Transduct Target Ther*. 2022 Jul 22;7(1):248. doi: 10.1038/s41392-022-01034-7.
